# *N*,*N′*,*N*′′-Tris(β-hydroxypropyl)hexahydro-1,3,5-triazine: MAK Value Documentation – Translation of the German version from 2023

**DOI:** 10.34865/mb2525450kske10_3or

**Published:** 2025-09-29

**Authors:** Andrea Hartwig, MAK Commission

**Affiliations:** 1 Institute of Applied Biosciences. Department of Food Chemistry and Toxicology. Karlsruhe Institute of Technology (KIT) Adenauerring 20a, Building 50.41 76131 Karlsruhe Germany; 2 Permanent Senate Commission for the Investigation of Health Hazards of Chemical Compounds in the Work Area. Deutsche Forschungsgemeinschaft, Kennedyallee 40, 53175 Bonn, Germany. Further information: Permanent Senate Commission for the Investigation of Health Hazards of Chemical Compounds in the Work Area | DFG

**Keywords:** N,N′,N′′-tris(β-hydroxypropyl)hexahydro-1,3,5-triazine, irritation, formaldehyde releaser, carcinogenicity, germ cell mutagenicity, sensitization, hydrolysis

## Abstract

The German Senate Commission for the Investigation of Health Hazards of Chemical Compounds in the Work Area (MAK Commission) has evaluated the data for *N*,*N*′,*N*′′-tris(β-hydroxypropyl)hexahydro-1,3,5-triazine [25254-50-6] to derive an occupational exposure limit value (maximum concentration at the workplace, MAK value) considering all toxicological end points. Relevant studies were identified from a literature search and also unpublished study reports were used. *N*,*N*′,*N*′′-Tris(β-hydroxypropyl)hexahydro-1,3,5-triazine is a formaldehyde releaser in aqueous solution. The substance is highly irritating to corrosive to the skin and eyes of rabbits. It is expected to undergo rapid hydrolysis in aqueous solution. For this reason, the observed local effects of irritation are attributed to the hydrolysis products formaldehyde and 1-aminopropan-2-ol. There are no studies that investigated the carcinogenic effects of *N*,*N*′,*N*′′-tris(β-hydroxypropyl)hexahydro-1,3,5-triazine and its toxicity and genotoxic potential in the upper respiratory tract or nose, which are assumed to be the likely target organs. The substance has low mutagenic and clastogenic potency in vitro, presumably due to the release of formaldehyde. Formaldehyde was classified in Carcinogen Category 4 because it causes tumours in nasal tissue at concentrations that exceed their detoxification capacity. As formaldehyde is released from *N*,*N*′,*N*′′-tris(β-hydroxpropyl)hexahydro-1,3,5-triazine, the substance could be classified in Carcinogen Caegory 4. However, because it is not possible to derive a MAK value for *N*,*N*′,*N*′′-tris(β-hydroxypropyl)hexahydro-1,3,5-triazine, the substance has been assigned to Carcinogen Category 2 and given the footnote “Prerequisite for Category 4 in principle fulfilled, but insufficient data available for the establishment of a MAK or BAT value”. As there are no data on the systemic bioavailability of *N*,*N*′,*N*′′-tris(β-hydroxypropyl)hexhydro-1,3,5-triazine and formaldehyde released by hydrolysis in tissues, there is no experimental evidence that the formaldehyde reaches the germ cells. Therefore, *N*,*N*′,*N*′′-tris(β-hydroxpropyl)hexahydro-1,3,5-triazine has been classified in Category 3 B for germ cell mutagens. The substance is a skin sensitizer in guinea pigs. Therefore, *N*,*N*′,*N*′′-tris(β-hydroxpropyl)hexahydro-1,3,5-triazine has been designated with “Sh” (for substances which cause sensitization of the skin). Skin contact is not expected to contribute sinificantly to systemic toxicity.

**Table:** 

**MAK value**	**–**
**Peak limitation**	**–**
	
**Absorption through the skin**	**–**
**Sensitization (2022)**	**Sh**
**Carcinogenicity (2022)**	**Category 2** ^a)^
**Prenatal toxicity**	**–**
**Germ cell mutagenicity (2022)**	**Category 3 B**
	
**BAT value**	**–**
	
Synonyms	hexahydro-1,3,5-tris(2-hydroxypropyl)-*s*-triazine α,α′,α′′-trimethyl-1,3,5-triazine-1,3,5(2H,4H,6H)triethanol reaction products of paraformaldehyde and 1-aminopropan-2-ol (2-hydroxypropylamine) (ratio 1:1)
Chemical name (IUPAC)	1-[3,5-bis(2-hydroxypropyl)-1,3,5-triazinan-1-yl]propan-2-ol
CAS number	25254-50-6
Structural formula	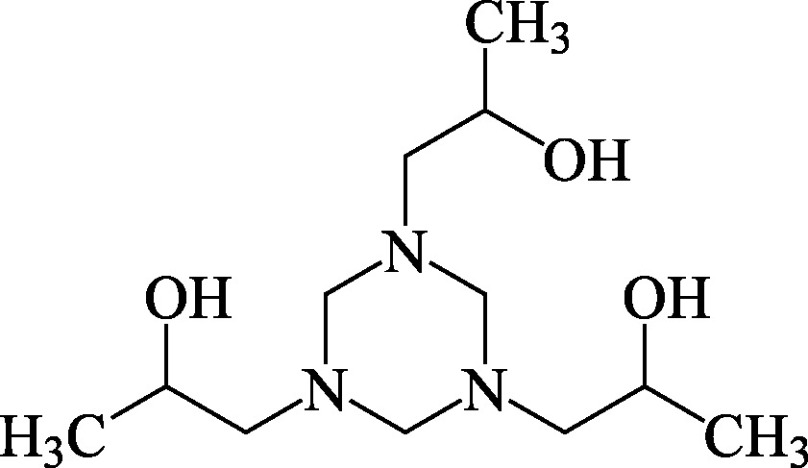
Molecular formula	C_12_H_27_N_3_O_3_
Molar mass	261.36 g/mol
Melting point	–36 to –38 °C (ECHA 2017)
Boiling point at 1013 hPa	40–195 °C (ECHA 2017)
Density at 20 °C	1.09–1.11 g/cm^3^ (ECHA 2017)
Vapour pressure	1.7 × 10^–8^ hPa (median, calculated) (US EPA 2022)
log K_OW_	cannot be determined due to hydrolysis (ECHA 2017)–0.019 (median, calculated) (US EPA 2022)–0.3 (calculated) (NCBI 2022)
Solubility	miscible with water (ECHA 2017)
pH	no data
pKa value	no data
	
Hydrolytic stability	rapid hydrolysis in water to formaldehyde and 1-aminopropan-2-ol (half-life < 1 hour). Due to strong dilution in water-miscible metal-working fluid (0.15%), complete hydrolysis to formaldehyde and 1-aminopropan-2-ol is to be expected. In metal-working fluid concentrate, incomplete hydrolysis can be assumed (ECHA 2017)
Stability	decomposes at 195 °C (ECHA 2017)
Production	*N,N′,N*′′-tris(β-hydroxypropyl)hexahydro-1,3,5-triazine is produced from paraformaldehyde and 1-aminopropan-2-ol in a ratio of 1:1 (Fraunhofer ITEM 2008)
Purity	it is a UVCB substance, a mixture whose qualitative or quantitative composition is unknown or variable. The ingredients are dependent on the concentration of the active substance, the temperature and the pH (ECHA 2017). The active substance is specified by the manufacturing process (no other details), in this case by the ratio of paraformaldehyde and 1-aminopropan-2-ol (1:1) and the definition of a formaldehyde content of 26%–30%, typically 28% (ECHA 2014)
Impurities	no data
Uses	disinfection during metal-working, preservation of fuels, preservation of closed water cooling systems and water-based cutting oils in metal-working (ECHA 2017)
Concentrations used	concentration in metal-working fluid concentrate: 3%; in water-miscible metal-working fluid: 0.1% to max. 0.2% (ECHA 2014, 2017)

^a)^ prerequisite for Category 4 in principle fulfilled, but insufficient data available for the establishment of a MAK or BAT value

Note: releases formaldehyde.

This documentation is based mainly on the publicly available data (ECHA 2014, 2017) pertaining to authorization in accordance with the Biocides Regulation. Cited unpublished toxicological studies from companies have been made available to the Commission.

## 1 Toxic Effects and Mode of Action

*N*,*N*′,*N*′′-Tris(β-hydroxypropyl)hexahydro-1,3,5-triazine releases formaldehyde in aqueous solution. The substance is highly irritating to corrosive to the skin and eyes of rabbits. In studies with single and repeated oral doses in rats, the irritant effects dominate. Rapid hydrolysis is to be expected in aqueous solution; the observed local irritation can be attributed, therefore, to the hydrolysis products formaldehyde and 1-aminopropan-2-ol, both of which are strong irritants.

*N*,*N*′,*N*′′-Tris(β-hydroxypropyl)hexahydro-1,3,5-triazine is a skin sensitizer in guinea pigs.

There are no developmental toxicity studies available.

The substance is clastogenic in mammalian cells in vitro, which is shown by the induction of chromosomal aberrations and also the formation of small colonies in the TK^+/–^ gene mutation test. The mutagenic effect in bacteria cannot be unequivocally evaluated. In mouse bone marrow, intraperitoneal injections of *N*,*N*′,*N*′′-tris(β-hydroxypropyl)hexahydro-1,3,5-triazine doses up to 100 mg/kg body weight did not induce micronuclei, but led to an increased number of chromosomal aberrations. A further chromosomal aberration test in the bone marrow after oral administration of up to 425 mg/kg body weight in mice yielded negative results. However, the maximum tolerated dose (MTD) was not reached in this test. Carcinogenicity studies with *N*,*N*′,*N*′′-tris(β-hydroxypropyl)hexahydro-1,3,5-triazine are not available.

## 2 Mechanism of Action

*N*,*N*′,*N*′′-Tris(β-hydroxypropyl)hexahydro-1,3,5-triazine releases formaldehyde in aqueous solution. This is the reason for its biocidal effect on bacteria and fungi (ECHA 2017).

The second hydrolysis product, 1-aminopropan-2-ol, is an amino alcohol that occurs naturally in the urine of humans and rats, as it is formed endogenously as a precursor for the biosynthesis of vitamin B12 (Greim 1994, available in German only).

### 2.1 Irritation

The local effects after oral administration and the irritant effect on skin and eyes are probably due to the released formaldehyde and 1-aminopropan-2-ol, both of which are highly irritating to corrosive (ECHA 2017), see MAK documentation and addenda on formaldehyde (Greim 2002; Hartwig 2014) and 1-aminopropan-2-ol (Greim 1994).

### 2.2 Genotoxic effects

The genotoxic effects of *N*,*N*′,*N*′′-tris(β-hydroxypropyl)hexahydro-1,3,5-triazine are probably due solely to formaldehyde (Greim 2002) as 1-aminopropan-2-ol is neither mutagenic nor clastogenic in mammalian cells in vitro (see ECHA 2020; Greim 1994). However, formaldehyde has more pronounced mutagenic effects in Salmonella typhimurium (Greim 2002) than *N*,*N*′,*N*′′-tris(β-hydroxypropyl)hexahydro-1,3,5-triazine. This is presumably due to the fact that formaldehyde formed from *N*,*N*′,*N*′′-tris(β-hydroxypropyl)hexahydro-1,3,5-triazine is not released abruptly in the medium and thus detoxification takes place simultaneously.

The in vivo tests yielded negative or questionably positive results. In all 3 studies it is unclear whether the MTD or the bone marrow was reached (see Section 5.6.2). It is also unclear whether, after the administration of formaldehyde, cytogenetic effects can only occur as a result of local exposure or also as a result of systemic availability of formaldehyde (Greim 2002). The negative in vivo results thus do not contradict those for formaldehyde.

## 3 Toxicokinetics and Metabolism

There are no studies available for the toxicokinetics and metabolism of *N*,*N*′,*N*′′-tris(β-hydroxypropyl)hexahydro-1,3,5-triazine. Both in vitro and in vivo, with the increasing dilution of the aqueous medium the substance is hydrolysed in increasing amounts to formaldehyde and 1-aminopropan-2-ol. The rate of hydrolysis and the equilibrium concentration are dependent on the concentration of the solution, the pH and the temperature (ECHA 2014, 2017; Fraunhofer ITEM 2008).

In a hydrolysis study according to OECD Test Guideline 111, a half-life could not be calculated for *N*,*N*′,*N*′′-tris(β-hydroxypropyl)hexahydro-1,3,5-triazine because at the earliest determination after 20 minutes 80% to 90% of the parent substance had already been hydrolysed (Fraunhofer ITEM 2008). A half-life of less than 20 minutes can therefore be assumed.

According to the results of the hydrolysis study, which was carried out in a closed system with a 1% solution, a maximum formaldehyde concentration of around 20% based on the initial weight of *N*,*N*′,*N*′′-tris(β-hydroxypropyl)hexahydro-1,3,5-triazine can be expected at a pH of 7 (Fraunhofer ITEM 2008). The proportion of formaldehyde in the entire molecule is 34.5%. In vivo, however, the degradation of formaldehyde and the metabolically released formic acid lowers the pH, thereby removing formaldehyde and the alkaline 1-aminopropan-2-ol from the equilibrium of hydrolysis. The resulting change in the concentration equilibrium creates an environment that enables faster splitting-off of formaldehyde. It can therefore be assumed that more than 20% formaldehyde is probably released in vivo.

The half-life of formaldehyde due to enzymatic degradation in the blood is about 1–1.5 minutes (EFSA 2014). With a long half-life of *N*,*N*′,*N*′′-tris(β-hydroxypropyl)hexahydro-1,3,5-triazine, the parallel detoxification of the resulting formaldehyde could protect against its adverse effects. The data from the hydrolysis study indicate rapid hydrolysis of *N*,*N*′,*N*′′-tris(β-hydroxypropyl)hexahydro-1,3,5-triazine, but data for the rate of hydrolysis in the first 15 minutes are not available. Therefore, for the worst case risk assessment it is assumed that the degradation of the formaldehyde formed from *N*,*N*′,*N*′′-tris(β-hydroxypropyl)hexahydro-1,3,5-triazine is slower than its formation.

There are no experimental studies available for percutaneous absorption. For a substance concentration of 1% in aqueous solution (in analogy to *N*,*N*′,*N*′′-tris(β-hydroxy**ethyl**)hexahydro-1,3,5-triazine, see Hartwig and MAK Commission 2025) under standard conditions (60 minutes exposure duration, 2000 cm^2^ of exposed skin) using a log K_OW_ of –0.019, model calculations according to IH SkinPerm v2.04 (Tibaldi et al. 2014) and Fiserova-Bergerova et al. (1990) yield an absorbed amount of about 18 and 54 μg/kg body weight, respectively (with log K_OW_ –0.3: 14 and 33 µg/kg body weight, respectively). The presumed rapid hydrolysis of the substance in the acidic environment of the skin’s surface, which counteracts the absorption of the undecomposed compound, was not taken into account.

## 4 Effects in Humans

There are no data available for *N*,*N*′,*N*′′-tris(β-hydroxypropyl)hexahydro-1,3,5-triazine in humans.

Unlike for the structurally similar *N*,*N*′,*N*′′-tris(β-hydroxy**ethyl**)hexahydro-1,3,5-triazine (Greim 2003, available in German only), a commercial test preparation with which the allergenic effects of *N*,*N*′,*N*′′-tris(β-hydroxypropyl)hexahydro-1,3,5-triazine could be investigated is not available. Therefore, there are no data available to date. However, due to the close structural relationship with *N*,*N*′,*N*′′-tris(β-hydroxy**ethyl**)hexahydro-1,3,5-triazine, which is known to cause skin sensitization in humans, and due to the hydrolytic formation of formaldehyde, sensitization of the skin in humans can likewise be expected with *N*,*N*′,*N*′′-tris(β-hydroxypropyl)hexahydro-1,3,5-triazine.

## 5 Animal Experiments and in vitro Studies

### 5.1 Acute toxicity

#### 5.1.1 Inhalation

There are no data available.

#### 5.1.2 Oral administration

In a study carried out according to OECD Test Guideline 401, the oral LD_50_ of *N*,*N*′,*N*′′-tris(β-hydroxypropyl)hexahydro-1,3,5-triazine in Wistar rats was 960 mg/kg body weight. Lethargy, abdominal breathing, wheezing and piloerection were observed after administration of the substance. Gross pathological examination revealed congestion and erosion of the mucosa in the glandular stomach, congestion and mucus secretion in the small intestine, emphysema and congestion in the lungs and marbled liver. Body weight gains were slightly decreased (ECHA 2014).

#### 5.1.3 Dermal application

A limit test according to OECD Test Guideline 402 in 5 male and 5 female Wistar rats resulted in a dermal LD_50_ of more than 2000 mg/kg body weight for *N*,*N*′,*N*′′-tris(β-hydroxypropyl)hexahydro-1,3,5-triazine. The animals exhibited mild to well-defined erythema. Three of the 10 rats developed hardening, dark discoloration and desquamation of the skin, which were not completely reversible by the end of the 14-day observation period. One animal with severe necrosis died after exposure to 2000 mg/kg body weight (ECHA 2014).

Another study carried out according to OECD Test Guideline 402 in Wistar rats likewise yielded an LD_50_ of more than 2000 mg/kg body weight. Neither mortality nor clinical signs were observed. No skin reactions were reported, except in 1 animal which was found to have localized skin defects and scarring on gross examination (ECHA 2014).

### 5.2 Subacute, subchronic and chronic toxicity

#### 5.2.1 Inhalation

There are no data available.

#### 5.2.2 Oral administration

In a 14-day range-finding study, groups of 5 male and 5 female Wistar rats were given daily gavage doses of* N*,*N*′,*N*′′-tris(β-hydroxypropyl)hexahydro-1,3,5-triazine of 0, 50, 100 or 200 mg/kg body weight and day in arachis oil. One male animal in the middle dose group died following dyspnoea and a swollen abdomen. In the high dose group, 1 female animal likewise exhibited dyspnoea, a poor general condition and reduced body weight. At 200 mg/kg body weight and day, the body weight gains of the female animals were reduced by about 10%, and their food intake was reduced. Based on these data, the authors selected doses of 12, 30, 80 and 200 mg/kg body weight and day for a subchronic study (ECHA 2014).

In another 14-day range-finding study, groups of 5 male and 5 female Wistar rats were given daily gavage doses of *N*,*N*′,*N*′′-tris(β-hydroxypropyl)hexahydro-1,3,5-triazine of 0, 100, 250 or 400 mg/kg body weight and day in distilled water. At 400 mg/kg body weight and day, the animals of both sexes displayed piloerection, lethargy and abdominal breathing. One female of the middle dose group died. Body weight gains and food intake were not significantly altered. There was only a slight reduction in both parameters in the male and female animals of the high dose group. The relative kidney weights were increased in the female animals at 250 mg/kg and above. Based on these data, the authors selected doses of 40, 100 and 250 mg/kg body weight and day for a subchronic study (ECHA 2014).

In a 90-day gavage study from 2002 in accordance with OECD Test Guideline 408, *N*,*N*′,*N*′′-tris(β-hydroxypropyl)hexahydro-1,3,5-triazine was administered daily by gavage to groups of 10 male and 10 female Wistar rats in doses of 0, 12, 30, 80 or 150 mg/kg body weight and day (0, 0.48%, 1.2%, 3.2% or 6% in arachis oil). The initial high dose was 200 mg/kg body weight and day. It was reduced to 150 mg/kg body weight and day from day 2 onwards due to severe clinical signs. According to the authors, the deaths of 3 female animals in the dose groups 12, 30 and 80 mg/kg body weight and day were not treatment-related (systemic arteritis). At 30 mg/kg body weight and day, 1 female animal died after bleeding from the nose, reduced skin turgor, bloody eyes and conjunctivitis. Examination revealed an enlargement of the submandibular lymph node in this animal. At 80 mg/kg body weight and day, 1 female animal with respiratory rales, reduced activity and bleeding from the nose died, as did 1 male animal (presumably due to a gavage error). In the high dose group, 2 animals died after the first dose (200 mg/kg body weight). However, these animals exhibited lesions in the pharynx and larynx, so that these deaths could also be due to errors in gavage administration. Two further animals died in the high dose group at later time points (no other details). At 30 mg/kg body weight and day, motor activity was reduced in 1 male and 1 female animal. At 80 mg/kg body weight and day, respiratory rales were heard in 3 males and 1 female, and in the high dose group 2 males and 2 females exhibited poor general condition, reduced activity and respiratory rales. A further 4 males and 3 females in the high dose group exhibited sporadic respiratory rales and 1 female reduced motor activity. Body weight gains were not significantly reduced in the males of the high dose group in weeks 7 to 13 (9%–10%). In addition, the food intake of these animals was slightly reduced. Gross examination revealed slight discoloration of the liver in 1 male at 12 mg/kg body weight and day, 1 male and 1 female at 30 mg/kg body weight and day, 4 males and 1 female at 80 mg/kg body weight and day and 2 males and 1 female at 150 mg/kg body weight and day. Eosinophilic cytoplasm of the hepatocytes was observed in the high dose group (n = 6) and at 80 mg/kg body weight (n = 2). In addition, glycogen vacuoles were absent in the hepatocytes mainly of males in the high dose group (n = 3). Single cell necrosis was observed in the liver of 4 male animals at 80 mg/kg body weight and day, but not in the high dose group. At 150 mg/kg body weight and day, oesophageal inflammation occurred in 3 of the 9 female animals studied and oesophageal myopathy in 1. According to the authors, the LOAEL (lowest observed adverse effect level) was 80 mg/kg body weight and day based on mortality, respiratory rales and findings in the larynx and pharynx; the effects on the liver were not considered relevant for the assessment (no other details). The NOAEL (no observed adverse effect level) was 30 mg/kg body weight and day (ECHA 2014). In aqueous solution, rapid hydrolysis of *N*,*N*′,*N*′′-tris(β-hydroxypropyl)hexahydro-1,3,5-triazine is to be expected; the local irritant effect observed is thus attributable to the hydrolysis products formaldehyde (Greim 2002; Hartwig 2014) and 1-aminopropan-2-ol (Greim 1994), both of which have a strong irritant effect. Some of the effects could also be due to the gavage treatment with arachis oil, which leads to the formation of an oil film in the oesophagus.

A second 90-day gavage study from 2002 according to OECD Test Guideline 408 was performed with 10 male and 10 female Wistar rats per dose group. The animals were given *N*,*N*′,*N*′′-tris(β-hydroxypropyl)hexahydro-1,3,5-triazine in daily doses of 0, 40, 100 or 250 mg/kg body weight and day (0, 0.4%, 1%, 2.5% in distilled water). There was an additional control and high dose group, which were kept without treatment for another 28 days after dosing. No treatment-related clinical signs occurred in the study. In the FOB (functional observational battery) examination at the end of the exposure period, the female animals in the high dose group exhibited a significant decrease in motor activity in the first 10 minutes and a significant reduction in total, motor and stereotypic activity in the second 10 minutes of the observation interval. At the end of the recovery period, motor activity was unchanged. During the last 3 weeks of the exposure period, body weight gains were reduced by about 9% in the males of the high dose group compared with the values for the controls, as well as in the females of the middle and high dose groups (about 8%). A statistically significant reduction in food intake was observed in the males of the middle and high dose groups only in week 11. The males in the high dose group were found to have a significantly reduced platelet count, which was, however, within the range of the historical control values. In the female animals of the high dose group, a statistically significant reduction in the MCV (mean corpuscular volume) was found and, in the recovery group, reduced MCHC (mean corpuscular haemoglobin concentration) and monocyte counts, and increased erythrocyte, haemoglobin, haematocrit and neutrophil counts. According to the authors (without further explanation), the values are not of toxicological relevance. In the male animals, the concentrations of sodium in the blood were increased even at the lowest dose, those of calcium at and above 100 mg/kg body weight and those of phosphorus at 250 mg/kg body weight. In the male animals, statistically significant increases were observed for the relative adrenal and testicular weights at 250 mg/kg body weight and for relative liver and heart weights at 100 and 250 mg/kg body weight. After the recovery phase, a statistically significant increase in the relative weights of the adrenal glands, epididymis and liver was still observed in the high dose group. In the female animals, the absolute adrenal weights were increased with statistical significance at 100 and 250 mg/kg body weight, as were the relative weights in all dose groups. The relative kidney weights were increased with statistical significance at 100 and 250 mg/kg body weight. According to the study authors, the NOAEL was 40 mg/kg body weight and day. The authors note that there was no histopathological correlate to the organ weight findings. In addition, predominantly the relative organ weights were affected, which may be the result of the reduced body weights. Also, despite the strong irritant properties of the substance, no effects on the gastrointestinal tract were found (ECHA 2014).

Both 90-day study reports are not available in the original and a conclusive evaluation is therefore not possible.

#### 5.2.3 Dermal application

There are no data available.

### 5.3 Local effects on skin and mucous membranes

#### 5.3.1 Skin

Three female New Zealand White rabbits were tested for skin irritation according to OECD Test Guideline 404. One animal was exposed semi-occlusively to 0.5 ml undiluted *N*,*N*′,*N*′′-tris(β-hydroxypropyl)hexahydro-1,3,5-triazine at 3 different application sites for 3 minutes, 1 hour and 4 hours respectively, while 2 other animals were exposed only for 4 hours. When the skin was assessed after 4 hours, well-defined erythema was observed at the sites with 3-minute and 1-hour exposure. The 4-hour exposure resulted in an irritation index (averaged over 24, 48 and 72 hours) of 2.55 for erythema and 2.0 for oedema on a scale with a maximum of 4.0. The individual values for erythema (24, 48 and 72 hours after the end of exposure) were 1.67, 2.33 and 3.67 and those for oedema 1.33, 2.00 and 2.67, respectively. In two of the 3 animals, the erythema was not reversible within the 14-day follow-up period, which indicates deeper skin damage (ECHA 2014).

A second skin irritation test according to OECD Test Guideline 404 was carried out in 3 male New Zealand White rabbits which were exposed semi-occlusively to 0.5 ml *N*,*N*′,*N*′′-tris(β-hydroxypropyl)hexahydro-1,3,5-triazine for 4 hours. At the end of the treatment, the test substance remaining on the skin was removed with water. The irritation index for erythema and oedema was calculated in this study after 24 and 72 hours and was 2.7 of a maximum of 4.0. Scab formation was observed in 2 of 3 animals after 7 days. All findings were reversible after 14 days (ECHA 2014).

In summary, *N*,*N*′,*N*′′-tris(β-hydroxypropyl)hexahydro-1,3,5-triazine is highly irritating to corrosive to rabbit skin.

#### 5.3.2 Eyes

Three New Zealand White rabbits were tested for eye irritation according to OECD Test Guideline 405. An amount of 0.1 ml undiluted *N*,*N*′,*N*′′-tris(β-hydroxypropyl)hexahydro-1,3,5-triazine was instilled into one eye and rinsed after 24 hours. Readings were taken 1 hour, 24, 48 and 72 hours and 7, 14 and 21 days after instillation. The mean irritation scores calculated by the authors were 2.33, 3.67, 4.67, 4.67, 3.67, 4.33 and 2.00, respectively. The damage to the cornea as well as redness and swelling of the conjunctivae were not reversible within 72 hours. The damage to the cornea of increasing severity that was not reversible up to 21 days after exposure (irritation scores: 1, 1, 1 of a maximum of 4, 72 hours after treatment; irritation scores: 1, 2, 2 of a maximum of 4, 21 days after treatment) is consistent with the fact that the substance releases formaldehyde (ECHA 2014).

In summary, *N*,*N*′,*N*′′-tris(β-hydroxypropyl)hexahydro-1,3,5-triazine is highly irritating to corrosive to the rabbit eye.

### 5.4 Allergenic effects

#### 5.4.1 Sensitizing effects on the skin

Two different maximization tests are available. One maximization test was performed in 20 female Dunkin-Hartley guinea pigs according to OECD Test Guideline 406. Intradermal induction was carried out with a 1% solution of the test substance, followed by topical induction with a 25% solution. Challenge treatment after 3 weeks with 5% and 10% solutions of the test substance caused slight irritation in all control animals. After a further 4 weeks, in a second challenge treatment with lower concentrations (1% and 2.5%) no skin effects were observed in any of the control animals, but 19 of the 20 pretreated animals reacted. Alembicol D (mainly triglycerides of the C8 and C10 fatty acids of coconut oil) was used as the vehicle (ECHA 2014).

A second maximization test with *N*,*N*′,*N*′′-tris(β-hydroxypropyl)hexahydro-1,3,5-triazine was performed in 10 male and 10 female Hartley guinea pigs according to OECD Test Guideline 406. After intradermal induction with a 1% solution of the test substance (vehicle: distilled water) and after topical induction and subsequent challenge treatment with undiluted test substance, 8 of the treated animals reacted (ECHA 2014). However, the undiluted substance was slightly irritating in the pre-test and strongly irritating to corrosive in rabbits (see Section 5.3.1). It is therefore unclear to what extent these reactions are due to the sensitizing or irritant effects. This positive test result is therefore not included in the evaluation.

In summary, *N*,*N*′,*N*′′-tris(β-hydroxypropyl)hexahydro-1,3,5-triazine and the structural analogue *N*,*N*′,*N*′′-tris(β-hydroxyethyl)hexahydro-1,3,5-triazine (Greim 2003) cause skin sensitization in guinea pigs.

Irrespective of the question of whether the sensitizing effects are due to the effects of the molecule as a whole or to the products formed by hydrolysis, the formaldehyde released by hydrolysis is to be regarded as causative at least to a large extent.

#### 5.4.2 Sensitizing effects on the airways

There are no data available.

### 5.5 Reproductive and developmental toxicity

#### 5.5.1 Fertility

There are no generation studies available.

The two 90-day studies in rats (see Section 5.2.2) did not reveal any effects of *N*,*N*′,*N*′′-tris(β-hydroxypropyl)hexahydro-1,3,5-triazine on the reproductive organs (ECHA 2014).

#### 5.5.2 Developmental toxicity

There are no data available.

### 5.6 Genotoxicity

#### 5.6.1 In vitro

A mutagenicity test in the Salmonella typhimurium strains TA98, TA100, TA102, TA1535 and TA1537 from the year 2000 was carried out according to OECD Test Guideline 471 with *N*,*N*′,*N*′′-tris(β-hydroxypropyl)hexahydro-1,3,5-triazine concentrations of 0, 18.7, 37.5, 75, 150 or 300 μg/plate. Neither in the presence nor in the absence of a metabolic activation system was there an increase in the number of revertants. In the pre-test, the next-higher concentration of 625 μg/plate was cytotoxic in the presence and absence of a metabolic activation system. The main test therefore did not test concentrations up to cytotoxic levels (ECHA 2014). The test was performed without preincubation, which, according to OECD Test Guideline 471, is the more sensitive test method for aldehydes.

A second mutagenicity test from 2000 was carried out with the Salmonella typhimurium strains TA98, TA100, TA1535, TA1537 and Escherichia coli WP2uvrA^–^. The concentrations used in the first experiment were 0, 5, 15, 50, 150, 500 or 1500 μg/plate for the Salmonella strains and 0, 15, 50, 150, 500, 1500 or 5000 μg/plate for Escherichia coli, each in the presence and absence of a metabolic activation system. In a second experiment, concentrations of 0, 5, 15, 50, 150, 300 or 500 μg/plate were used for the strain TA100 with the addition of a metabolic activation system. Without the addition of a metabolic activation system, *N*,*N*′,*N*′′-tris(β-hydroxypropyl)hexahydro-1,3,5-triazine was not mutagenic in any of the strains. In the presence of a metabolic activation system, a concentration-dependent mutagenic effect was observed in strain TA100. The increase in the revertant count was less than twofold, but according to the authors of the registration dossier the number of revertants was significantly higher than that of the historical controls, so that the substance was regarded as weakly mutagenic. A cytotoxic effect was observed at 500 μg/plate and above, both with and without the addition of a metabolic activation system (ECHA 2014). The test was performed without preincubation, which, according to OECD Test Guideline 471, is the more sensitive test method for aldehydes.

A chromosomal aberration test from 2001 was performed in CHL (Chinese hamster lung) cells according to OECD Test Guideline 473. *N*,*N*′,*N*′′-Tris(β-hydroxypropyl)hexahydro-1,3,5-triazine was tested without the addition of a metabolic activation system at concentrations of 0, 3.6, 7.3, 14.5, 22 or 29 μg/ml (29 μg/ml cytotoxic) and with the addition of a metabolic activation system at concentrations of 0, 7.3, 14.5, 22, 29, 58, 87 or 116 μg/ml for 6 hours, followed by an 18-hour post-incubation period. A concentration-dependent clastogenic effect and the induction of polyploidy were observed without the addition of a metabolic activation system at and above 7.3 μg/ml and with the addition of a metabolic activation system (no concentration specified). Predominantly chromosome breaks and exchanges were observed. The mitotic index was reduced to 80% at 7.3 µg/ml without the addition of a metabolic activation system (ECHA 2014).

In a TK^+/–^ mutation assay in L5178Y mouse lymphoma cells carried out according to OECD Test Guideline 476, *N*,*N*′,*N*′′-tris(β-hydroxypropyl)hexahydro-1,3,5-triazine yielded positive results with and without the addition of a metabolic activation system. The cells were incubated for 3 hours with concentrations of 0, 2.5, 5, 10, 20, 40 or 60 μg/ml (without the addition of a metabolic activation system) and with 0, 5, 10, 20, 40, 60 or 80 μg/ml (with the addition of a metabolic activation system). The maximum test concentrations were determined in a pre-test due to the onset of cytotoxicity at higher concentrations. The relative growth of the cells was determined in the main study. *N*,*N*′,*N*′′-Tris(β-hydroxypropyl)hexahydro-1,3,5-triazine induced an increased number of mutants in a concentration-dependent and statistically significant manner. The predominantly small colonies are evidence of clastogenic effects and the reason for the positive test results. The relative growth of the cells at the lowest mutagenic concentration (10 μg/ml without or 20 μg/ml with the addition of a metabolic activation system) was greater than 80% (ECHA 2014).

A second TK^+/–^ mutation test in L5178Y mouse lymphoma cells carried out according to OECD Test Guideline 476 confirmed the result. *N*,*N*′,*N*′′-Tris(β-hydroxypropyl)hexahydro-1,3,5-triazine likewise induced a concentration-dependent and statistically significant increase in the number of mutants and predominantly small colonies in the presence and absence of a metabolic activation system. The cells were incubated for 3 hours with concentrations of 0, 2.5, 5, 10, 20, 30 or 40 μg/ml. The maximum test concentrations were determined in a pre-test due to the onset of cytotoxicity at higher concentrations. The relative growth of the cells was determined in the main study. The relative growth of the cells was greater than 80% at the lowest mutagenic concentration of 20 μg/ml (ECHA 2014).

#### 5.6.2 In vivo

A chromosomal aberration test from 2002 carried out in the bone marrow of mice according to OECD Test Guideline 475 revealed a weak clastogenic effect of *N*,*N*′,*N*′′-tris(β-hydroxypropyl)hexahydro-1,3,5-triazine. The substance was administered intraperitoneally to 5 male and 5 female NMRI mice in single doses of 0, 10, 50 or 100 mg/kg body weight (in 0.9% NaCl in water) and the bone marrow cells of the animals were examined for chromosomal aberrations 24 hours later (in animals of the high dose group additionally after 48 hours). The number of aberrations, predominantly chromosome breaks and fragments, in the treated male and female animals was increased approximately 2 to 4.5-fold compared with that in the control animals. Polyploid cells were not increased in a statistically significant manner compared with the control values. No clinical signs occurred at any of the doses in the main study. The doses were selected after a preliminary study in which a female mouse was treated intraperitoneally with the single dose of 200 mg/kg body weight and displayed partial paralysis, dyspnoea and apathy immediately after treatment. Apathy occurred also in 3 male and 3 female animals that received a single intraperitoneal dose of 100 mg/kg body weight. According to the study authors, the result is inconclusive as there was a high standard deviation and the results were only slightly higher than those in the historical controls (0.0%–4.5% aberrant cells). However, the study authors did not discuss the results of the concurrent positive and negative controls. According to the authors of the biocide dossier, there is evidence of a clastogenic effect of *N*,*N*′,*N*′′-tris(β-hydroxypropyl)hexahydro-1,3,5-triazine (ECHA 2014).

A second chromosomal aberration test from 2000 according to OECD Test Guideline 475 was carried out after oral administration of 0, 106, 212 or 425 mg/kg body weight twice within 24 hours in groups of 5 male and 5 female Swiss mice. The high dose of the study corresponded to 60% of the LD_50_ of 710 mg/kg body weight. The bone marrow of the animals was examined 24 hours after the last treatment. There were no clinical signs or effects on body weights. *N*,*N*′,*N*′′-Tris(β-hydroxypropyl)hexahydro-1,3,5-triazine did not cause a reduction in the mitotic index or the induction of chromosomal aberrations (ECHA 2014). However, the MTD was not reached and it cannot be conclusively determined whether the bone marrow was reached. The positive control indicated that the test system functioned correctly.

In a micronucleus test from 2002 carried out in accordance with OECD Test Guideline 474, 5 male and 5 female NMRI mice per group were given single intraperitoneal injections of 0, 10, 50 or 100 mg *N*,*N*′,*N*′′-tris(β-hydroxypropyl)hexahydro-1,3,5-triazine/kg body weight (in 0.9% NaCl in water). Examination took place after 24 hours and, for the high dose, also after 48 hours. In each case, 2000 polychromatic erythrocytes (PCE) per animal were analysed. The doses were selected after a preliminary study in which female mice were treated intraperitoneally with single doses of 200 mg/kg body weight and immediately afterwards paralysis, dyspnoea, apathy and closed eyes were observed in some animals. Apathy occurred also after the administration of 100 mg/kg body weight. This was also seen in the main study, in which the animals were apathetic and kept their eyes closed 1 hour after treatment with the high dose. *N*,*N*′,*N*′′-Tris(β-hydroxypropyl)hexahydro-1,3,5-triazine did not induce micronuclei in the bone marrow in this study. According to the study authors, the ratio of PCE to total erythrocytes was reduced at the high dose. However, according to the authors of the biocide dossier, the values are within the laboratory’s historical control values (n = 10) and it is not clear whether the MTD or the bone marrow was reached in the study (ECHA 2014).

#### 5.6.3 Summary

*N*,*N*′,*N*′′-Tris(β-hydroxypropyl)hexahydro-1,3,5-triazine is clastogenic in mammalian cells in vitro; this effect is based on chromosomal aberrations and evident in the formation of small colonies in the TK^+/–^ gene mutation test. Two mutagenicity tests in bacteria are available which, however, do not allow a clear evaluation due to various limitations (insensitive test system; negative results, but not tested to the maximum tolerated concentration; positive results, but the number of revertants less than twice the control value).

*N*,*N*′,*N*′′-Tris(β-hydroxypropyl)hexahydro-1,3,5-triazine hydrolyses completely to formaldehyde and 1-aminopropan-2-ol (see Section 1). Since 1-aminopropan-2-ol does not lead to mutagenic or clastogenic effects in mammalian cells in vitro (ECHA 2020; Greim 1994), it can be assumed that the positive results are due to the release of formaldehyde.

In the mouse bone marrow, intraperitoneal injections of *N*,*N*′,*N*′′-tris(β-hydroxypropyl)hexahydro-1,3,5-triazine doses up to 100 mg/kg body weight did not induce micronuclei, although it is unclear whether the MTD was reached. In an identical experiment, an increased number of chromosomal aberrations was found in the bone marrow of the mice; this result was evaluated as questionably positive. Another chromosomal aberration test with oral administration of up to 425 mg/kg body weight in mice yielded negative results. Here the MTD was not reached and it is not known whether the bone marrow was reached.

It is also unclear whether, after the administration of formaldehyde, cytogenetic effects can only occur as a result of local exposure or also as a result of systemic availability of formaldehyde (Greim 2002). The available negative test results in vivo thus do not contradict those for formaldehyde.

### 5.7 Carcinogenicity

There are no studies available for* N*,*N*′,*N*′′-tris(β-hydroxypropyl)hexahydro-1,3,5-triazine or the hydrolysis product 1-aminopropan-2-ol.

The local carcinogenicity of the second hydrolysis product formaldehyde is extensively documented (see ECHA 2014; Greim 2002; Hartwig 2014).

## 6 Manifesto (MAK value/classification)

Due to the hydrolysis product formaldehyde, *N*,*N*′,*N*′′-tris(β-hydroxypropyl)hexahydro-1,3,5-triazine can be assumed to have carcinogenic effects and cause local irritation. These are therefore also the most sensitive end points.

**Carcinogenicity. **There are no carcinogenicity studies available for *N*,*N*′,*N*′′-tris(β-hydroxypropyl)hexahydro-1,3,5-triazine. The substance has low mutagenic and clastogenic potency in vitro. This is presumably due to the release of formaldehyde. A possible genotoxic effect on the likely target organs upper respiratory tract and nose has not been investigated to date.

The local carcinogenicity of the hydrolysis product formaldehyde, on the other hand, is well documented (see ECHA 2014; Greim 2002; Hartwig 2014). Formaldehyde is classified in Carcinogen Category 4, as it is carcinogenic in nasal tissues at concentrations that exceed their detoxification capacity. *N*,*N*′,*N*′′-Tris(β-hydroxypropyl)hexahydro-1,3,5-triazine releases formaldehyde very rapidly in aqueous solution. Thus, 80% of the initial substance is hydrolysed within 20 minutes and the half-life in aqueous solution is less than 20 minutes; this could not be determined more precisely as shorter time points were not investigated. In a hydrolysis study according to OECD Test Guideline 111, about 20% formaldehyde is released from a 0.1% solution at pH 7 (Fraunhofer ITEM 2008). In vivo, the amount released is presumably higher, as the hydrolysis products are eliminated and thus shift the reaction equilibrium in the direction of hydrolysis, so that complete splitting-off of formaldehyde can be assumed (see Section 3). In addition, 1-aminopropan-2-ol has a local irritant effect. If the detoxification capacity for formaldehyde in the nose is not exceeded, no carcinogenic effects occur there. Due to the local carcinogenic effect of the hydrolysis product formaldehyde, *N*,*N*′,*N*′′-tris(β-hydroxypropyl)hexahydro-1,3,5-triazine could, by analogy, be classified in Carcinogen Category 4. However, as a MAK value cannot be derived for *N*,*N*′,*N*′′-tris(β-hydroxypropyl)hexahydro-1,3,5-triazine (see Section MAK value and peak limitation), the substance has been assigned to Carcinogen Category 2 and given the footnote “Prerequisite for Category 4 in principle fulfilled, but insufficient data available for the establishment of a MAK or BAT value”.

**MAK value and peak limitation. **There are no inhalation studies available for *N*,*N*′,*N*′′-tris(β-hydroxypropyl)hexahydro-1,3,5-triazine. The rate of hydrolysis of the substance depends on the concentration, the pH and the temperature, whereby 3 molecules each of formaldehyde and 1-aminopropan-2-ol can be formed from one molecule of *N*,*N*′,*N*′′-tris(β-hydroxypropyl)hexahydro-1,3,5-triazine.

In an inhalation study with the structurally similar *N*,*N*′,*N*′′-tris(β-hydroxy**ethyl**)hexahydro-1,3,5-triazine in rats (Hartwig 2015, available in German only), the stronger local irritant effect (LOAEC (lowest observed adverse effect concentration) 3 mg/m^3^ ≙ 0.33 ml/m^3^) after 28-day exposure compared with that of formaldehyde and 2-aminoethanol is probably the result of aerosol impaction. Due to the strong effects of *N*,*N*′,*N*′′-tris(β-hydroxy**ethyl**)hexahydro-1,3,5-triazine at the LOAEC of the 28-day study and the lack of a NOAEC (no observed adverse effect concentration), a MAK value could not be derived from this study (see Hartwig and MAK Commission 2025). Due to the role of aerosol impaction, a MAK value could not be established in analogy to that for gaseous formaldehyde (see documentation “*N*,*N*′,*N*′′-Tris(β-hydroxy**ethyl**)hexahydro-1,3,5-triazine”, Hartwig and MAK Commission 2025).

This can likewise be assumed for the *N*,*N*′,*N*′′-tris(β-hydroxypropyl)hexahydro-1,3,5-triazine evaluated here. The results of the vapour pressure measurements (6.4 × 10^–5^ hPa at 20 °C, 1.3 × 10^–4^ hPa at 25 °C (OECD TG 104; effusion method, vapour pressure equilibrium); 9.303 hPa at 25 °C (static method; ECHA 2014)) are probably subject to errors, as is the case with the structurally similar *N*,*N*′,*N*′′-tris(β-hydroxy**ethyl**)hexahydro-1,3,5-triazine. It is more likely that the vapour pressures of the two triazines are in a similar range and that the *N*,*N*′,*N*′′-tris(β-hydroxypropyl)hexahydro-1,3,5-triazine is still present as an aerosol at concentrations at which formaldehyde is already in vapour form. Due to the stronger effects of aerosol impaction, a MAK value cannot be derived for *N*,*N*′,*N*′′-tris(β-hydroxypropyl)hexahydro-1,3,5-triazine. Peak limitation is therefore not applicable.

When used in dilute aqueous solutions, complete hydrolysis should be expected and therefore the MAK value for formaldehyde (Greim 2002; Hartwig 2014) should be observed.

**Prenatal toxicity. **Assignment to a pregnancy risk group is not applicable, as no MAK value has been established.

**Germ cell mutagenicity. ***N*,*N*′,*N*′′-Tris(β-hydroxypropyl)hexahydro-1,3,5-triazine is clastogenic in mammalian cells in vitro, which is shown by the induction of chromosomal aberrations and the formation of small colonies in the TK^+/–^ gene mutation test. Its mutagenicity in bacteria cannot be unequivocally assessed. In mouse bone marrow, intraperitoneal doses of up to 100 mg *N*,*N*′,*N*′′-tris(β-hydroxypropyl)hexahydro-1,3,5-triazine/kg body weight did not induce micronuclei, although it is unclear whether the MTD was reached. In an identical experiment, an increased number of chromosomal aberrations occurred in the bone marrow of the mice; this result was evaluated as questionably positive due to the high standard deviations. A further chromosomal aberration test in the bone marrow after oral administration of up to 425 mg/kg body weight in mice yielded negative results. In this test, the MTD was not reached and it is not known whether the bone marrow was reached. Studies in germ cells are not available.

Since 1-aminopropan-2-ol is not mutagenic or clastogenic in mammalian cells in vitro (see ECHA 2020; Greim 1994), it can be assumed that the positive in vitro results of *N*,*N*′,*N*′′-tris(β-hydroxypropyl)hexahydro-1,3,5-triazine are due to the release of formaldehyde. Assuming that after inhalation all formaldehyde is rapidly released in the upper respiratory tract via hydrolysis, it is probably not systemically available. However, data for this are not available.

Formaldehyde is classified in Category 5 for germ cell mutagens. This means that, if the MAK value of 0.3 ml/m^3^ is observed, inhalation exposure to formaldehyde is not expected to make any significant contribution to the genetic risk for humans (Greim 2002; RAC and SEAC 2020). Theoretically, by analogy with formaldehyde, *N*,*N*′,*N*′′-tris(β-hydroxypropyl)hexahydro-1,3,5-triazine could be classified in Category 5 for germ cell mutagens. However, a MAK value cannot be established for *N*,*N*′,*N*′′-tris(β-hydroxypropyl)hexahydro-1,3,5-triazine. Since data for the systemic bioavailability of *N*,*N*′,*N*′′-tris(β-hydroxypropyl)hexahydro-1,3,5-triazine and the formaldehyde released by hydrolysis are not available, there is no experimental evidence that the released formaldehyde reaches the germ cells in active form. Therefore, *N*,*N*′,*N*′′-tris(β-hydroxypropyl)hexahydro-1,3,5-triazine has been classified in Category 3 B for germ cell mutagens.

**Absorption through the skin. **There are no experimental data available for the absorption of *N*,*N*′,*N*′′-tris(β-hydroxypropyl)hexahydro-1,3,5-triazine through the skin. Estimates based on model calculations yield a maximum absorbed amount of 54 μg/kg body weight. This is far below the systemic NOAEL of 40 mg/kg body weight for rats. For a person of 70 kg, the maximum total amount of *N*,*N*′,*N*′′-tris(β-hydroxypropyl)hexahydro-1,3,5-triazine expected to be absorbed under the named conditions is 3.78 mg or 0.014 mmol. Assuming rapid complete hydrolysis, this results in the release of 1.26 mg or 0.042 mmol formaldehyde. The physiological formaldehyde level in human blood is about 2–3 mg/l or 10–15 mg in 5 litres of blood (Heck et al. 1985). The maximum additional contribution of formaldehyde thus lies within the variation range of the physiological concentrations. Therefore, *N*,*N*′,*N*′′-tris(β-hydroxypropyl)hexahydro-1,3,5-triazine is not designated with an “H” (for substances which can be absorbed through the skin in toxicologically relevant amounts).

**Sensitization. **There are no findings of skin sensitization in humans, but a clearly positive result from a test in guinea pigs with the use of adjuvant. Despite the lack of clinical findings to date, but due to the ability of the substance to release formaldehyde and its close structural relationship to *N*,*N*′,*N*′′-tris(β-hydroxy**ethyl**)hexahydro-1,3,5-triazine, which is known to be a skin sensitizer in humans (Hartwig and MAK Commission 2025), *N*,*N*′,*N*′′-tris(β-hydroxypropyl)hexahydro-1,3,5-triazine has been designated with “Sh” (for substances which cause sensitization of the skin).

There are no studies available for sensitization of the airways. Therefore, *N*,*N*′,*N*′′-tris(β-hydroxypropyl)hexahydro-1,3,5-triazine has not been designated with “Sa” (for substances which cause sensitization of the airways).
